# Heart failure documentation in outpatients with diabetes and volume overload: an observational cohort study from the Diabetes Collaborative Registry

**DOI:** 10.1186/s12933-020-01190-6

**Published:** 2020-12-12

**Authors:** Suzanne V. Arnold, Philip G. Jones, Michael Beasley, Jeanine Cordova, Abhinav Goyal, Gregg C. Fonarow, Leo Seman

**Affiliations:** 1 Saint Luke’s Mid America Heart Institute and University of Missouri-Kansas City, 4401 Wornall Rd, Kansas City, MO 64111 USA; 2 Yale University School of Medicine, New Haven, CT USA; 3 Boehringer Ingelheim Pharmaceuticals, Ridgefield, CT USA; 4 Emory University School of Medicine, Atlanta, GA USA; 5 University of California, Los Angeles, Los Angeles, CA USA

**Keywords:** Heart failure, Diabetes, Quality of care

## Abstract

**Background:**

Heart failure is a common and devastating complication of type 2 diabetes (T2D). Prompt recognition of heart failure may avert hospitalization, facilitate use of guideline-directed therapies, and impact choice of T2D medications. We sought to determine the rate and factors associated with heart failure documentation in T2D patients with evidence of volume overload requiring loop diuretics.

**Methods:**

DCR is an on-going, prospective US registry of outpatient T2D patients from > 5000 cardiology, endocrinology, and primary care clinicians (current analysis used data from 2013–2019). Among T2D patients receiving loop diuretics, we examined the rate of chart documentation of heart failure. We used a 3-level hierarchical logistic regression model (patients nested within physician within practice) to examine factors associated with heart failure diagnosis.

**Results:**

Among 1,322,640 adults with T2D, 225,125 (17.0%) were receiving a loop diuretic, of whom 91,969 (40.9%) had documentation of heart failure. Male sex, lower body mass index, atrial fibrillation, chronic kidney disease, and coronary artery disease were associated with greater odds of heart failure diagnosis. After accounting for patient factors, patients seen by cardiologists were the most likely to have HF documented followed by PCPs and then endocrinologists.

**Conclusions:**

Among US outpatients with T2D, 17% of patients had evidence of volume overload—defined by loop diuretic prescription—of whom fewer than half had a clinical diagnosis of heart failure. While there may be non-heart failure indications for loop diuretics, our data suggest that a substantial proportion of T2D patients may have unrecognized heart failure and therefore could be missing opportunities for targeted therapies that could alter the clinical course of heart failure.

## Background

While heart failure (HF) and type 2 diabetes (T2D) are individually highly prevalent and morbid, the intersection of the two is becoming increasingly recognized as a major health concern. HF in patients with T2D is predominantly due to diastolic dysfunction [1], and may or may not be associated with concomitant coronary artery disease [2, 3]. Establishing a diagnosis of HF in patients with T2D is not only important from a prognosis perspective but could have important treatment implications. Use of guideline-directed medical therapy for HF (especially in the setting of left ventricular dysfunction) can substantially reduce the risk of hospitalization and mortality [4]. In addition, the presence or absence of HF has important implications in the management of T2D. For example, metformin [5, 6] and sodium–glucose co-transporter-2 (SGLT-2) inhibitors [7, 8] may be beneficial in patients with HF whereas thiazolidinediones [9, 10] and, possibly, some dipeptidyl peptidase-4 inhibitors [11, 12] should be avoided (or used with caution). As HF is often slowly progressive and symptoms such as dyspnea could be attributed to other conditions, recognizing HF in the ambulatory setting may be challenging, but early recognition could allow for appropriate medical therapy to be initiated prior to overt HF requiring hospitalization. In order to better define the potential gap in care from a lack of identification of co-morbid HF in outpatients with T2D, we used the Diabetes Collaborative Registry (DCR) to determine the rate and factors associated with HF diagnosis in T2D patients with evidence of volume overload requiring loop diuretics.

## Methods

### Patient population

DCR is a US-based, outpatient, quality improvement registry that began in 2014 (with data collected retrospectively from 2013 and available through Q1 2019 for this analysis) as a collaborative effort by endocrinology, primary care, and cardiology professional societies [13]. DCR was designed to understand the care of patients with diabetes across the spectrum of primary and specialty care and currently includes 379 practices (174 primary care, 182 cardiology, 23 endocrinology) and 5133 providers. Data are collected through an automated system integration solution that extracts relevant data elements from electronic health records (including patient demographics, comorbidities, and medications). This study was limited to adults with T2D, and the most recent clinic visit was used for analysis.

The primary outcome for this study was chart documentation of HF in patients with evidence of volume overload requiring prescription of loop diuretics. We acknowledge that use of loop diuretics is a moderate surrogate for diagnosis of HF, as (1) patients could have indolent HF that is not recognized and therefore not on loop diuretics and (2) patients may be on loop diuretics for another indication (e.g., hypertension with advanced chronic kidney disease). In a real-world registry without routine measurements of left ventricular pressures, however, we believe that the use of loop diuretics is the best surrogate available for volume overload, which in the majority of patients is indicative of some degree of HF. We excluded patients with chronic liver disease, as these patients could have volume overload without HF. Because participation in the registry requires no data collection beyond that of the routine clinical care and all collected data are de-identified, a waiver of written informed consent and authorization for this study was granted by Chesapeake Research Review Incorporated.

### Statistical analysis

Among patients on loop diuretics, demographic and clinical characteristics were compared between patients who did versus did not have a chart diagnosis of HF using standardized differences (> 10% difference is considered clinically relevant [14]). A 3-level hierarchical logistic regression model was used to examine the association of patient factors and provider specialty with documentation of HF. Patient factors included age, sex, race, body mass index, coronary artery disease, chronic kidney disease, and atrial fibrillation. Splines were included for continuous variables to explore non-linear associations. Provider and practice were both included as random effects to account for the clustering of patients within providers and also clustering of providers within practices. Total variability across providers was quantified by the median odds ratio (combining both the provider- and practice-level effects), which estimates the average relative difference in odds of documentation of HF between 2 different providers for patients with identical covariates. Missing covariate data were imputed using multiple imputation methods. The imputation model included all variables in the analytic model. Imputed values were obtained using random forests, as implemented in the R package ‘missRanger’ [15]. Twenty randomly imputed data sets were obtained, the above model was fit on all data sets, and model estimates were pooled using Rubin’s method to obtain final estimates of odds ratios and confidence intervals [16]. All analyses were performed with SAS version 9.4 (SAS Institute, Cary, North Carolina) and R version 3.6.3 (R Core Team, Vienna, Austria). All p-values are 2-sided tests and were considered statistically significant at < 0.05.

## Results

### Study cohort

There were 1,482,642 adults with T2D enrolled in DCR from 2013–2019, of whom 160,002 patients had chronic liver disease and were excluded from the analysis. Among the 1,322,640 remaining patients, 225,125 patients (17.0%) were prescribed a loop diuretic and formed the primary analytic cohort. Mean age was 70.6 ± 11.9 years, 0.752% were women, 55.3% had coronary artery disease, and 4.5% had chronic kidney disease (Table 1).

**Table 1:** Patient characteristics according to documentation of heart failure

	All Patients on loop diuretics n = 215,957	Heart failure documented n = 110,809	Heart failure not documented n = 105,148	Standardized difference^a^
Age (years)	70.6 ± 11.9	72.1 ± 11.8	69.5 ± 11.9	22.7%
Men	106,584/225,111 (47.3%)	48,867/91,965 (53.1%)	57,717/133,146 (43.3%)	19.7%
Race				4.4%
White	135,394/161,754 (83.7%)	54,130/65,360 (82.8%)	81,264/96,394 (84.3%)	
Black	23,239/161,754 (14.4%)	9804/65,360 (15.0%)	13,435/96,394 (13.9%)	
Other	3001/161,754 (1.9%)	1370/65,360 (2.1%)	1631/96,394 (1.7%)	
Multiracial	120/161,754 (0.1%)	56/65,360 (0.1%)	64/96,394 (0.1%)	
Body mass index (kg/m^2^)	34.4 ± 8.7 (n = 161,705)	33.3 ± 8.5 (n = 67,707)	35.2 ± 8.7 (n = 93,998)	21.8%
Current smoker	67,481/215,433 (31.3%)	28,506/88,703 (32.1%)	38,975/126,730 (30.8%)	10.2%
Hypertension	198,558 (88.2%)	83,614 (90.9%)	114,944 (86.3%)	14.5%
Dyslipidemia	170,251 (75.6%)	72,169 (78.5%)	98,082 (73.7%)	11.3%
Coronary artery disease	124,602 (55.3%)	64,937 (70.6%)	59,665 (44.8%)	54.1%
Prior myocardial infarction	26,765 (11.9%)	16,727 (18.2%)	10,038 (7.5%)	32.2%
Prior stroke	48,692 (21.6%)	23,257 (25.3%)	25,435 (19.1%)	14.9%
Atrial fibrillation/flutter	70,661 (31.4%)	41,917 (45.6%)	28,744 (21.6%)	52.5%
Chronic kidney disease	10,131 (4.5%)	4429 (4.8%)	5702 (4.3%)	2.6%
Systolic blood pressure (mmHg)	129.0 ± 18.4 (n = 211,968)	126.7 ± 18.7 (n = 87,160)	130.6 ± 18.0 (n = 124,808)	21.2%
Diastolic blood pressure (mmHg)	72.6 ± 10.8 (n = 211,781)	71.4 ± 11.0 (n = 87,079)	73.5 ± 10.7 (n = 124,702)	19.8%
LV function documented	97,728 (43.4%)	54,560 (59.3%)	43,168 (32.4%)	56.1%
LV function				67.6%
Hyperdynamic (> 70%)	5464/97,728 (5.6%)	2015/54,560 (3.7%)	3449/43,168 (8.0%)	
Normal (50–70%)	60,698/97,728 (62.1%)	28,108/54,560 (51.5%)	32,590/43,168 (75.5%)	
Mildly reduced (40–49%)	12,636/97,728 (12.9%)	8785/54,560 (16.1%)	3851/43,168 (8.9%)	
Moderately reduced (30–39%)	9606/97,728 (9.8%)	7762/54,560 (14.2%)	1844/43,168 (4.3%)	
Severely reduced (< 30%)	9324/97,728 (9.5%)	7890/54,560 (14.5%)	1434/43,168 (3.3%)	
Beta blocker	174,779 (77.6%)	80,424 (87.4%)	94,355 (70.9%)	41.7%
ACE inhibitor or ARB	166,536 (74.0%)	68,787 (74.8%)	97,749 (73.4%)	3.2%
Diabetes medications				
Insulin	81,480 (36.2%)	33,705 (36.6%)	47,775 (35.9%)	1.6%
Metformin	108,987 (48.4%)	41,894 (45.6%)	67,093 (50.4%)	9.7%
Sulfonylurea	66,620 (29.6%)	28,085 (30.5%)	38,535 (28.9%)	3.5%
Thiazolidinedione	14,920 (6.6%)	4732 (5.1%)	10,188 (7.7%)	10.3%
DPP-4 inhibitor	34,038 (15.1%)	13,149 (14.3%)	20,889 (15.7%)	3.9%
GLP-1 agonist	19,384 (8.6%)	5705 (6.2%)	13,679 (10.3%)	14.8%
SGLT-2 inhibitor	13,462 (6.0%)	4015 (4.4%)	9447 (7.1%)	11.8%

Data are presented as mean ± standard deviation, n (%), or n/N (%) if reported data are lower than column header

*LV* left ventricular, *ACE* angiotensin converting enzyme, *ARB* angiotensin II receptor blocker, *DPP* dipeptidyl peptidase, *GLP* glucagon-like peptide, *SGLT* sodium–glucose cotransporter

^a^> 10% is considered a clinical relevant difference [14]

### HF documentation

Among 225,125 patients who were prescribed loop diuretics, HF was documented in 91,969 patients (40.9%), and the demographics, comorbidities, and glucose-lowering medications of patients who did versus did not have HF documented are shown in Table 1. Patients with HF documented were more likely to have left ventricular function documented (HF documented vs. no: 59.3% vs. 32.4%, standardized difference 56.1%) and to be on beta blockers (87.4% vs. 70.9%, standardized difference 41.7%) but had similar use of ACE inhibitors/ARBs (74.8% vs. 73.4%, standardized difference 3.2%). Patients with HF documented were less likely to be treated with metformin (HF documented vs. no: 45.6% vs. 50.4%, standardized difference 9.7%), thiazolidinediones (5.1% vs. 7.7%, standardized difference 10.3%), GLP-1 receptor agonists (6.2% vs. 10.3%, standardized difference 14.8%), and SGLT2 inhibitors (4.4$ vs. 7.1%, standardized difference 11.8%).

**Fig. 1:**
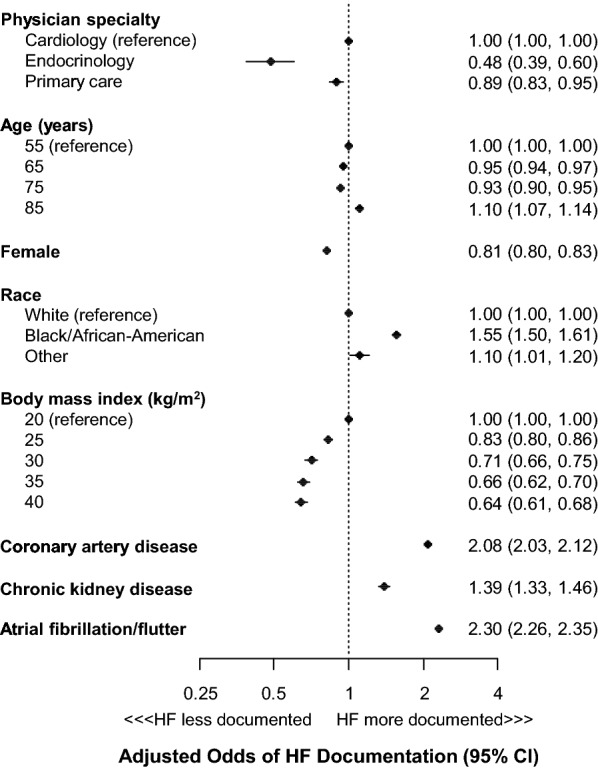
Association of patient factors and specialty on documentation of heart failure in T2D patients on loop diuretics

**Fig. 2:**
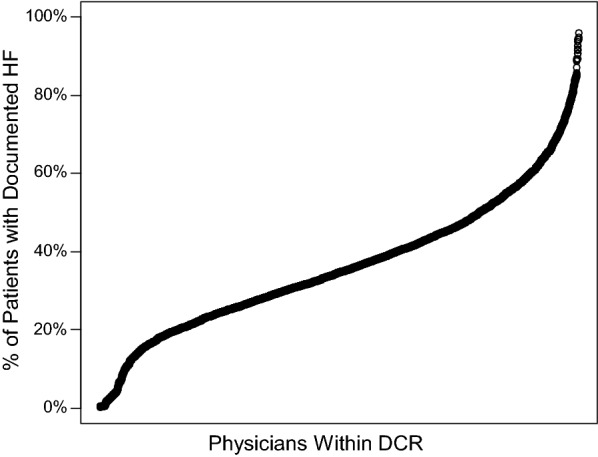
Physician-level variability in documentation of heart failure. Each circle represents an individual physician in DCR and the percentage of patients that physician saw who were on loop diuretics and had heart failure documented. The x-axis indicates physicians in DCR ordered by percentage of patients with heart failure documented

In the hierarchical logistic regression model, female sex and higher body mass index were independently associated with a lower odds of HF documentation (Fig. 1). Black race, coronary artery disease, chronic kidney disease, and atrial fibrillation were each associated with a greater odds of HF documentation. After accounting for patient factors, patients seen by cardiologists were the most likely to have HF documented followed by primary care physicians and then endocrinologists (Additional file 1: Table S1). There was also substantial variation across providers in whether or not HF was documented, with probabilities of documentation ranging from near 0% to near 100% (Fig. 2). The median odds ratio was 2.50, indicating that, for two randomly selected providers of the same specialty seeing patients with identical covariates, there is 50% chance of a greater than 2.5-fold difference in the odds of HF documentation between the two providers.

## Discussion

In a large US database of adults with T2D from cardiology, endocrinology, and primary care outpatient practices, 17% had evidence of volume overload requiring prescription of loop diuretics, of whom over half did not have HF documented. Patients who were female, obese, and who did not have cardiovascular diseases were less likely to have HF documented. Furthermore, even after accounting for differences in patient characteristics, there was substantial variation across providers in the documentation of HF, suggesting that some providers are more diligent at recognizing and documenting HF in patients with evidence of volume overload. Documentation appeared to impact cardiac testing (evaluation of left ventricular function), use of beta blockers, and choice of glucose-lowering medications, with lower use metformin and SGLT2 inhibitors (paradoxically, given their benefit in patients with HF [5, 17–19], prescription patterns that have been previously noted [1]) and thiazolidinediones in patients in whom HF was documented. These data suggest that a substantial proportion of T2D patients may have unrecognized HF and could be missing opportunities for targeted therapies that could impact outcomes [4, 5, 7].

### Implications

HF is a particularly morbid condition, increasing the risk of hospitalization [20–22], increasing healthcare costs [23], and worsening patients’ quality of life [24]. Volume overload that leads to hospitalization typically prompts laboratory and imaging tests that permit a straightforward diagnosis of HF. However, the subtler signs and symptoms of indolent HF may be overlooked in the ambulatory setting, with the dyspnea and exercise intolerance attributed to obesity, deconditioning, or age. Furthermore, as HF in patients with T2D is more likely to occur with preserved ejection fraction [1] and often occurs in the absence of coronary disease [3], common triggers for a HF diagnosis are often absent. Nonetheless, prompt recognition of HF in the ambulatory office could influence choice of glucose-lowering medications and potentially trigger cardiology referral, laboratory or imaging tests, and closer monitoring of volume status—all of which could help avert HF hospitalization and improve outcomes [25, 26]. Furthermore, if the prompted imaging tests demonstrate reduced left ventricular ejection fraction, a number of evidence-based treatments could be instituted to improve outcomes (e.g., angiotensin converting enzyme-inhibitors, beta blockers, defibrillators). Greater recognition of the high prevalence of HF as well as the differences in presentation of HF in patients with T2D (more HF with preserved ejection fraction, less associated with coronary disease) may help providers more consistently diagnose HF in patients with T2D and evidence of volume overload.

### Limitations

First and most importantly, we used prescription of loop diuretics as a surrogate marker for HF, but this may under- or over-estimate the proportion of patients with clinical HF. As mentioned above, dyspnea with more subtle volume overload due to HF may be falsely attributed to obesity, deconditioning, lung disease, or age, and therefore may not treated with diuretics. Furthermore, patients could have HF that is managed through salt and fluid restriction and not require diuretics for management (e.g., 36% of patients in DCR with left ventricular dysfunction were not on loop diuretics [27]). Conversely, patients may not have HF and still be treated with loop diuretics [appropriately or inappropriately (e.g., venous insufficiency)]. There are a few indications for loop diuretics other than volume overload (hypertension with advanced chronic kidney disease, nephrotic syndrome, cirrhosis), although these impact a fairly limited proportion of patients. As such, without comprehensive testing of left ventricular pressures, we cannot be certain of the true prevalence of HF in ambulatory patients with T2D. Second, it is possible that HF was recognized by the provider but not documented in the electronic health record. This is particularly an issue with endocrinologists who may be uncomfortable assigning a diagnosis of HF, which is considered outside of their specialty. Third, other than some evidence of an impact on choice of glucose-lowering medications, we cannot determine if there is any clinical consequence of a lack of HF documentation in patients on loop diuretics. Further longitudinal work is needed to define the association of a lack of HF documentation with incident HF hospitalization or other outcomes (e.g., costs, mortality).

## Conclusion

In a large US cohort of outpatients with T2D, 17% of patients had evidence of volume overload requiring prescription of loop diuretics, fewer than half of whom had documentation of HF. While there may be non-HF indications for loop diuretics, our data suggest that a substantial proportion of patients with T2D have unrecognized HF and may be missing targeted interventions that could potentially alter the clinical course of HF and reduce the risk of hospitalization and mortality.

## Supplementary Information

**Additional file 1: Table S1.** Patient characteristics according to documentation of heart failure, stratified by physician specialty.

## Data Availability

The data that support the findings of this study are available from the Diabetes Collaborative Registry, but restrictions apply to the availability of these data, which were used under specified agreement for the current study, and so are not publicly available. Data are however available from the authors upon reasonable request and with permission of DCR.

## Abbreviations

HF: Heart failure
T2D: Type 2 diabetes
SGLT2: Sodium–glucose co-transporter-2
DCR: Diabetes Collaborative Registry
