# Supplementary material for: Heart failure documentation in outpatients with diabetes and volume overload: an observational cohort study from the Diabetes Collaborative Registry
Source: Cardiovasc Diabetol. 2020 Dec 12;19:212. doi: 10.1186/s12933-020-01190-6 (PMC7733267; doi:10.1186/s12933-020-01190-6)
Supplement: Supplementary file 1 — Additional file 1: Table S1. Patient characteristics according to documentation of heart failure, stratified by physician specialty. [file 12933_2020_1190_MOESM1_ESM.docx]

| **Table S1. Patient characteristics according to documentation of heart failure, stratified by physician specialty** | | | | | | | | |
| --- | --- | --- | --- | --- | --- | --- | --- | --- |
|  | **Cardiology (n=106593)** | | | **Endocrinology (n=13597)** | | | **Primary Care (n=103,662)** | |
|  | **Heart Failure Documented** | **Heart Failure Not Documented** | **Heart Failure Documented** | | **Heart Failure Not Documented** | **Heart Failure Documented** | | **Heart Failure Not Documented** |
|  | **n=53,873** | **n=52,720** | **n=1405** | | **n=12,192** | **n=36,209** | | **n=67,453** |
| Age (years) | 72.2 ± 11.6 | 70.8 ± 11.4 | 69.2 ± 11.5 | | 68.0 ± 11.6 | 72.1 ± 12.1 | | 68.6 ± 12.2 |
| Men | 29486/53870 (54.7%) | 24851/52712 (47.1%) | 695/1405 (49.5%) | | 5056/12191 (41.5%) | 18423/36208 (50.9%) | | 27480/67452 (40.7%) |
| Race |  |  |  | |  |  | |  |
| White | 29499/36352 (81.1%) | 28823/34891 (82.6%) | 791/901 (87.8%) | | 6655/7909 (84.1%) | 23541/27729 (84.9%) | | 45271/52975 (85.5%) |
| Black | 5940/36352 (16.3%) | 5257/34891 (15.1%) | 93/901 (10.3%) | | 1134/7909 (14.3%) | 3699/27729 (13.3%) | | 6949/52975 (13.1%) |
| Other | 875/36352 (2.4%) | 788/34891 (2.3%) | 16/901 (1.8%) | | 114/7909 (1.4%) | 472/27729 (1.7%) | | 720/52975 (1.4%) |
| Multiracial | 38/36352 (0.1%) | 23/34891 (0.1%) | 1/901 (0.1%) | | 6/7909 (0.1%) | 17/27729 (0.1%) | | 35/52975 (0.1%) |
| Body mass index (kg/m^2^) | 33.1 ± 8.3 (n=40357) | 34.6 ± 8.4 (n=39223) | 34.8 ± 8.5 (n=733) | | 35.9 ± 8.5 (n=6273) | 33.6 ± 8.8 (n=26223) | | 35.6 ± 8.9 (n=47918) |
| Current smoker | 14395/52089 (27.6%) | 12188/50374 (24.2%) | 353/1340 (26.3%) | | 3025/11653 (26.0%) | 13598/34809 (39.1%) | | 23527/63951 (36.8%) |
| Hypertension | 48932 (90.8%) | 47610 (90.3%) | 1192 (84.8%) | | 9052 (74.2%) | 33045 (91.3%) | | 57580 (85.4%) |
| Dyslipidemia | 42487 (78.9%) | 39785 (75.5%) | 1146 (81.6%) | | 9056 (74.3%) | 28152 (77.7%) | | 48661 (72.1%) |
| Coronary artery disease | 40986 (76.1%) | 33347 (63.3%) | 695 (49.5%) | | 2476 (20.3%) | 22901 (63.2%) | | 23543 (34.9%) |
| Prior myocardial infarction | 10899 (20.2%) | 6014 (11.4%) | 180 (12.8%) | | 279 (2.3%) | 5570 (15.4%) | | 3703 (5.5%) |
| Prior stroke | 13882 (25.8%) | 12555 (23.8%) | 255 (18.1%) | | 953 (7.8%) | 8973 (24.8%) | | 11749 (17.4%) |
| Atrial fibrillation/flutter | 25564 (47.5%) | 16133 (30.6%) | 371 (26.4%) | | 767 (6.3%) | 15765 (43.5%) | | 11726 (17.4%) |
| Chronic kidney disease | 2174 (4.0%) | 1541 (2.9%) | 141 (10.0%) | | 1305 (10.7%) | 2102 (5.8%) | | 2839 (4.2%) |
| Systolic blood pressure (mmHg) | 126.8 ± 18.8 (n=51603) | 130.9 ± 18.8 (n=50787) | 125.8 ± 15.9 (n=1319) | | 128.6 ± 15.9 (n=11142) | 126.5 ± 18.5 (n=33776) | | 130.6 ± 17.6 (n=62141) |
| Diastolic blood pressure (mmHg) | 71.2 ± 11.0 (n=51530) | 72.9 ± 10.8 (n=50706) | 71.7 ± 9.8 (n=1316) | | 73.0 ± 9.6 (n=11133) | 71.6 ± 11.1 (n=33771) | | 74.1 ± 10.7 (n=62126) |
| LV function documented | 40790 (75.7%) | 32764 (62.2%) | 111 (7.9%) | | 159 (1.3%) | 13397 (37.0%) | | 10066 (14.9%) |
| LV function |  |  |  | |  |  | |  |
| Hyperdynamic (>70%) | 1581/40790 (3.9%) | 2695/32764 (8.2%) | 2/111 (1.8%) | | 7/159 (4.4%) | 420/13397 (3.1%) | | 727/10066 (7.2%) |
| Normal (50-70%) | 21161/40790 (51.9%) | 25147/32764 (76.8%) | 49/111 (44.1%) | | 101/159 (63.5%) | 6757/13397 (50.4%) | | 7206/10066 (71.6%) |
| Mildly reduced (40-49%) | 6498/40790 (15.9%) | 2890/32764 (8.8%) | 14/111 (12.6%) | | 19/159 (11.9%) | 2232/13397 (16.7%) | | 928/10066 (9.2%) |
| Moderately reduced (30-39%) | 5805/40790 (14.2%) | 1233/32764 (3.8%) | 24/111 (21.6%) | | 20/159 (12.6%) | 1892/13397 (14.1%) | | 586/10066 (5.8%) |
| Severely reduced (<30%) | 5745/40790 (14.1%) | 799 (2.4%) | 22/111 (19.8%) | | 12/159 (7.5%) | 2096/13397 (15.6%) | | 619/10066 (6.1%) |
| Beta blocker | 47730 (88.6%) | 41108 (78.0%) | 1239 (88.2%) | | 8256 (67.7%) | 31037 (85.7%) | | 44460 (65.9%) |
| ACE inhibitor or ARB | 40530 (75.2%) | 39603 (75.1%) | 1118 (79.6%) | | 9231 (75.7%) | 26786 (74.0%) | | 48335 (71.7%) |
| *Diabetes medications* |  |  |  | |  |  | |  |
| Insulin | 20299 (37.7%) | 18569 (35.2%) | 975 (69.4%) | | 7731 (63.4%) | 12272 (33.9%) | | 21182 (31.4%) |
| Metformin | 24926 (46.3%) | 27212 (51.6%) | 777 (55.3%) | | 6700 (55.0%) | 16012 (44.2%) | | 32865 (48.7%) |
| Sulfonylurea | 17323 (32.2%) | 16530 (31.4%) | 529 (37.7%) | | 4109 (33.7%) | 10106 (27.9%) | | 17699 (26.2%) |
| Thiazolidinedione | 3007 (5.6%) | 4105 (7.8%) | 135 (9.6%) | | 1500 (12.3%) | 1565 (4.3%) | | 4551 (6.7%) |
| DPP-4 inhibitor | 8000 (14.8%) | 8288 (15.7%) | 332 (23.6%) | | 2712 (22.2%) | 4754 (13.1%) | | 9776 (14.5%) |
| GLP-1 agonist | 3067 (5.7%) | 3593 (6.8%) | 394 (28.0%) | | 3318 (27.2%) | 2228 (6.2%) | | 6697 (9.9%) |
| SGLT-2 inhibitor | 2146 (4.0%) | 2481 (4.7%) | 263 (18.7%) | | 2273 (18.6%) | 1591 (4.4%) | | 4659 (6.9%) |

LV, left ventricular; ACE, angiotensin converting enzyme; ARB, angiotensin II receptor blocker; DPP, dipeptidyl peptidase; GLP, glucagon-like peptide; SGLT, sodium-glucose cotransporter
